# Supplementary material for: Sensitive Electrochemical Non-Enzymatic Detection of Glucose Based on Wireless Data Transmission
Source: Sensors (Basel). 2022 Apr 5;22(7):2787. doi: 10.3390/s22072787 (PMC9003393; doi:10.3390/s22072787)
Supplement: Supplementary file 1 [file sensors-22-02787-s001.zip › sensors-1632094-supplementary.pdf]

sensors

Supplementary Materials

Article

Sensitive Electrochemical Non-Enzymatic Detection of Glucose Based on Wireless Data Transmission

Young-Joon Kim [1], Somasekhar R. Chinnadayyala [2], Hien T. Ngoc Le [1,*] and Sungbo Cho [1,3,*]

[1] Department of Electronic Engineering, Gachon University, 1342 Seongnam-daero, Seongnam 13120, Korea; youngkim@gachon.ac.kr
[2] Sensors and Aerosols Laboratory, Department of Mechanical Engineering, Ulsan National Institute of Science and Technology (UNIST), Ulsan 44919, Korea; ssreddy@unist.ac.kr
[3] Gachon Advanced Institute for Health Science & Technology, Gachon University, 155 Gaetbeol-ro, Incheon 21999, Korea
* Correspondence: ltnh1809@gachon.ac.kr (H.T.N.L.); sbcho@gachon.ac.kr (S.C.)

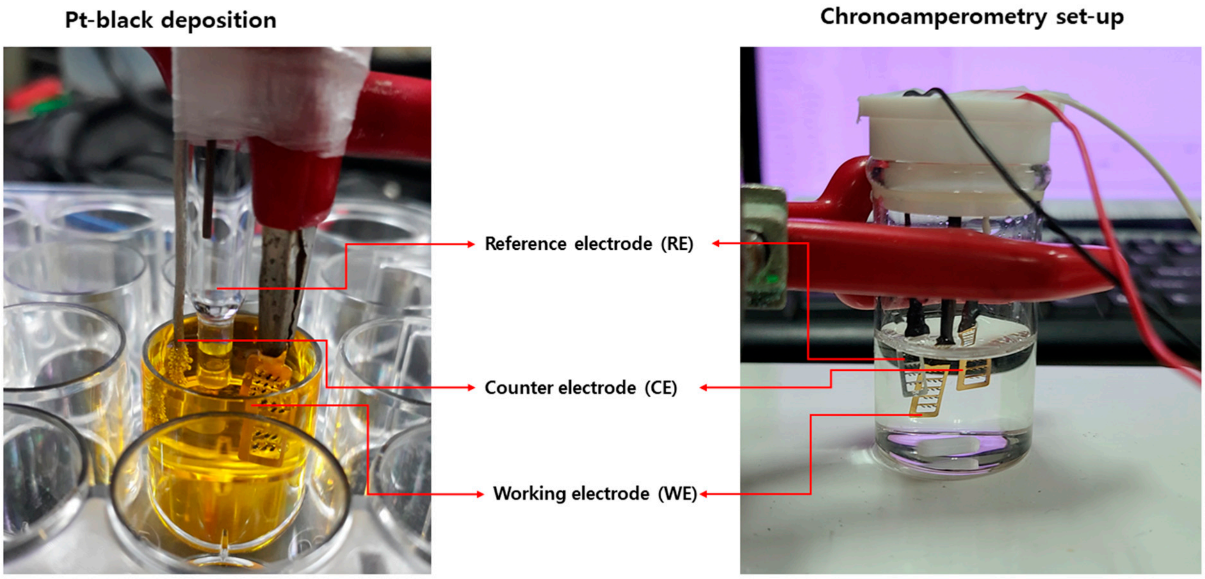

Figure S1. Electrochemical deposition of Pt-black by potentiometry in a three-electrode configuration: using bare gold microneedle as the working electrode (WE), platinum wire as the counter electrode (CE) and Ag/AgCl as the external reference electrode (RE). Electrodeposition was carried out at −2.5 mA cm^{-2} for 200 s (**a**). Constant potential chronoamperometric experimental setup for in vitro non-enzymatic glucose determination at an applied potential of +0.12 V vs. AgCl MN in a three-electrode configuration using Ab/Pt-black/Nf microneedle as the working electrode (WE), Au/Pt-black as the counter electrode and AgCl MN as reference electrode (RE). The electrode response was measured after the stabilization of the background currents, and glucose was spiked at an interval of 50 s (**b**).

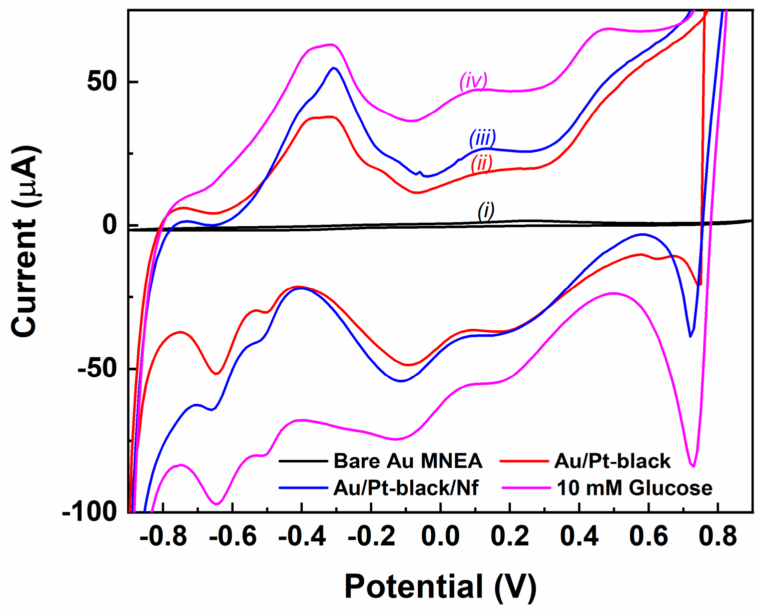

Figure S2. Cyclic voltammetry (CV) of the bare Au MNEA (i), Au/Pt-black (ii) and Au/Pt-black/Nf (iii) with 10 mM Glucose addition (iv) at a scan rate of 50 mV s^{-1} in 10×PBS (pH = 7.4).
